# Sheffield and the Red Cross Grant

**Published:** 1919-10-11

**Authors:** 


### Sheffield and the Red Cross Grant.

TEXT OF THE CORRESPONDENCE.

At the recent quarterly meeting of the Board of Sheffield Royal Infirmary Mr. H. Bedford said that they would have noticed that no grant had been received from the Red Cross surplus lately distributed to the voluntary hospitals. He wrote " rather a strong letter " to the Secretary to urge the claims of the institution. The following reply had been received from the Territorial Branch of the St. John Ambulance Association, West Riding Section, York :

" I am quite aware of the excellent work that has been done for the soldiers during the war by the Royal Infirmary, and also that they most kindly, at my request, admitted some very difficult cases of paralysis, free of charge.

" The opinion prevailing at the B.R.C.S. is that Sheffield is a very wealthy city, and that many of its inhabitants have made large fortunes during the war. It is, therefore, probable that the inhabitants are in a position to support their own institutions. I think, therefore, the question of a grant can only be considered if the appeal that you are about to make does not meet with a very large amount of local support. Kindly let me hear from you further on this point.

Yours faithfully,

C. W. E. Duncombe,

*County Director.*"

Mr. Bedford thought they would be able to answer that letter satisfactorily. Where big fortunes had been made in Sheffield during the war the Government had taken 80 per cent. of the excess profits, and there had been other taxes, which left things very little different from what they were when on a pre-war level.

The people in London were looking to the Sheffield people to help this institution, and, on the whole, the state of the fund was satisfactory, but they would like to get the £100,000 before the year was out. £87,000 has been promised.

A Consultative Advisory Council has held its first meeting under Sir Henry Hadow, Vice-Chancellor of the University, with representatives from the committees of the different hospitals and the Edgar Allen Institute. Both lay and medical members of each institution are on the Board, also representatives of the University and three Labour members.

